# HIV and Zika: When will we be able to end these epidemics?

**DOI:** 10.1186/s12977-016-0315-4

**Published:** 2016-11-15

**Authors:** Mark A. Wainberg, Andrew M. L. Lever

**Affiliations:** 1 McGill University AIDS Centre, Jewish General Hospital, Montreal, QC Canada; 2 Cambridge University, Cambridge, UK

It seems that a year cannot go by without some new emergent infectious disease stealing away headlines from the HIV epidemic, even though the latter continues to kill several million people each year. For example, there was justifiable concern that the recent Ebola epidemic would become more widespread than it did [1]. Fortunately, Ebola seems to have receded, at least for now, the epidemic being declared over in January 2016, although precautions must continue to be taken to ensure that it does not again become a threat.

This year, the world has been thrown into panic by Zika virus that has caused hundreds of infants to be born with a condition termed microcephaly. The reasons for this terrible birth defect are not well understood and it seems that it is the northeast area of Brazil that has been most prone to this devastating complication. Hopefully, new epidemiological data will shed light on the reasons that microcephaly has not occurred to nearly the same extent in other parts of Brazil or in Colombia and other countries where transmission of Zika has taken place on a large scale.

We need to be justly concerned about the mosquito-borne transmission of flaviviruses in general and of Zika, dengue, and Chikungunya viruses in particular, since the latter are now endemic in many parts of the Caribbean, Africa, India, and elsewhere. And we should also hope that the novel hormonally-based strategies aimed at sterilization of the *Aedes aegypti* mosquitos that transmit these viruses will be successful.

Perhaps it is not surprising, given their transmission routes and acuity compared to HIV, that multiple differences exist between the flaviviruses and HIV in terms of strategies aimed at control of these diseases. The most important medical advances in regard to HIV have undoubtedly been in the area of antiretroviral (ARV) drug development, and there is little doubt that the ARVs have saved the lives of millions of people around the world [2]. At the same time ARVs have also proven useful in pre-exposure prophylaxis (PrEP), which is a concept whereby the same drugs that work in treatment can also be used to reduce the likelihood of infection [3, 4]. Furthermore, the development of ARVs has also spawned the principle known as Treatment as Prevention, whereby it is hoped that the successful treatment of HIV-infected persons will lower their levels of plasma viremia to non-detectability, with the consequence that successfully-treated persons will no longer be infectious for their sexual contacts [5]. These advances have even propelled the World Health Organization to propose a plan termed ‘90–90–90’ aimed at ending the HIV epidemic by the year 2030. This concept articulates that we will identify 90% of the world’s HIV infected population, and then go on to use ARVs to treat 90% of those who test positive, and thereby successfully reduce viral load to non-detectability in 90% of the latter.

Although most observers agree that these are laudable goals, they question whether the identification of 90% of the world’s HIV infected subjects will be possible when estimates suggest that 40% of infected people are unaware of their HIV status and it is known, for example, that many persons who are at high risk for acquisition of HIV are often themselves reluctant to take an HIV test. Perhaps we will need to find ways of incentivizing people to be tested for HIV if we are to achieve the first 90 in the WHO scheme [6]. It will probably also be necessary to find ways of incentivizing care-givers as well so that they routinely offer the test and “insist” that it be used.

In contrast, most observers agree that the best way to control Zika, dengue and Chikungunya probably revolves around the development of safe and effective preventive vaccines. For example, how could we contemplate using a potential new anti-Zika drug to treat infected pregnant women, if we did not know with certainty that such a drug did not itself cause birth defects? The same can probably be said for drugs that might block the replication of dengue and Chikungunya, although there might be rationale for the use of such products in non-pregnant populations, and, of course, the use of antiviral compounds might help to limit transmission from humans to *Aedes aegypti* mosquitos and help to break the chain of epidemic transmission. Hopefully, vaccines and related approaches such as the use of passively transfused antibodies will soon be available to help control Zika infection. Thus, the approaches that need to be developed to arrest flavivirus transmission may differ significantly from those that have been used to interfere with transmission of HIV.

The tragedy of Zika for a pregnant mother, her affected infant and the wider family is not something to dismiss lightly. It is good news that this is a relatively uncommon complication but it is no less terrible for that. At the same time, we should not lose sight of the fact that the worldwide burden of HIV disease continues to expand, in part because so many people have been saved by ARVs. It is estimated that as many as 38 million people worldwide are now living with HIV. Sadly, a number almost equal to this are thought to already have died of HIV infection, making the epidemic one of the worst to have ever affected humankind. There are still several million new HIV cases occurring annually, and the problem of HIV drug-resistance in developing country settings is on the rise and poses a real threat to the success of the WHO 90–90–90 goal to end the epidemic. On World AIDS Day, December 1, let us turn our attention with renewed energy toward finding ways of ending the HIV epidemic as well as other infectious diseases that continue to take a horrible toll, often of the most vulnerable and disadvantaged.
